# Characterizing the Effects of Intermittent Faults on a Processor for Dependability Enhancement Strategy

**DOI:** 10.1155/2014/286084

**Published:** 2014-04-28

**Authors:** Chao(Saul) Wang, Zhong-Chuan Fu, Hong-Song Chen, Dong-Sheng Wang

**Affiliations:** ^1^Multicore Research Institute, High Performance CPU Center, Tsinghua University, Building F.I.T, Beijing 100084, China; ^2^School of Computer Science, Harbin Institute of Technology, No. 155 Fanrong Street, Nangang District, Harbin 150001, China; ^3^School of Computer and Communication Engineering, University of Science and Technology Beijing, No. 30 Xueyuan Road, Haidian District, Beijing 100083, China

## Abstract

As semiconductor technology scales into the nanometer regime, intermittent faults have become an increasing threat. This paper focuses on the effects of intermittent faults on NET versus REG on one hand and the implications for dependability strategy on the other. First, the vulnerability characteristics of representative units in OpenSPARC T2 are revealed, and in particular, the highly sensitive modules are identified. Second, an arch-level dependability enhancement strategy is proposed, showing that events such as core/strand running status and core-memory interface events can be candidates of detectable symptoms. A simple watchdog can be deployed to detect application running status (IEXE event). Then SDC (silent data corruption) rate is evaluated demonstrating its potential. Third and last, the effects of traditional protection schemes in the target CMT to intermittent faults are quantitatively studied on behalf of the contribution of each trap type, demonstrating the necessity of taking this factor into account for the strategy.

## 1. Introduction

Semiconductor technology scaling into the nanometer regime has impelled a resurgence of interest in intermittent faults. The driving forces include shrinking geometries, smaller interconnect dimensions, lower power voltages, and decreased noise margins, all of which have a negative impact on the dependability of circuits under transient, permanent, and, in particular, intermittent faults [1, 2]. In addition, it is forecast that multicore is more vulnerable to intermittent faults in future technology [3].

Unlike transient faults, intermittent faults occur in bursts. Also, in contrast to permanent faults, they arise only in particular situations and do not persist. The following characteristics distinguish intermittent from transient and permanent faults.

- Burst. Intermittent faults occur in bursts whose duration can vary across a wide range of timescales from orders of cycles to even milliseconds or more.
- Nonrepeatability. Intermittent faults (e.g., caused by defects) are expected to arise under particular situations (e.g., elevated temperature, voltage droops, etc.).
- Fixed Location. Once activated, intermittent faults repeatedly occur at the same location or from the same module of a processor. Consequently, replacement of the offending component eliminates intermittent faults, which is in contrast to transient faults which cannot be fixed by repair [4].

Above all, intermittent faults are expected to become more frequent in the nanometer regime and have become an increasing threat to multicore.

The above distinguishing features and the complicated source of failures (SOFs) of intermittent faults leave many uncertainties to be exploited. To the best of our knowledge, we are the first to adopt a SPARC T2 chip multithreading (CMT) processor as a case study to characterize the fault effects. Thus, a dependability enhancement strategy is proposed. This paper focuses on fault effects on NET versus REG on one hand and the implications for dependability enhancement strategy on the other. Major contributions are as follows.

*First*, a detailed evaluation of the vulnerability characteristics is made using sensitivity metrics. The target CMT is exercised with two workloads, featuring memory intensive and CPU-intensive, respectively. A similar trend in the effect of intermittent faults is revealed, and, in particular, the common highly sensitive modules are identified. This corroborates that the susceptible characteristics do not vary with workloads in terms of sensitivity metric [5].

*Second*, through a thorough breakdown of the outcome categories, a novel light-weight arch-level dependability enhancement strategy is proposed, showing that core/strand running status and core-memory interface events can be candidates of the detectable symptoms across all the modules under investigation (*DeadLock* and* Invalid Packet* in this paper). Application running status (*incomplete execution, IEXE* event) can be covered by a simple watchdog to further refine the proposed light-weight arch-level strategy and the silent data corruption (SDC) is estimated demonstrating its potential.

*Third*, to the best of our knowledge we are the first to make a quantitative study of the effect of traditional protection schemes in the target CMT in terms of the contribution of each trap type, showing the necessity of taking this factor into account for the strategy [6].

In Section 2, we describe experimental methodology.  Section 3 makes a thorough investigation of the vulnerability characteristics by sensitivity metrics. Then, Section 4 prospects an arch-level dependability enhancement strategy against intermittent faults and the SDC is evaluated demonstrating its potential.  Section 5 discusses the protection effect of traditional schemes in the target CMT, including ECC and parity. Related work is described in Section 6, and Section 7 provides a conclusion.

## 2. Experimental Methodology

### 2.1. Target System

The target system is a CMT version of the UltraSPARC processor. Representative units are selected as device under test (DUT), including (1) address generation unit (AGEN) in instruction fetch unit (IFU), (2) pick unit (PKU), (3) decoder, (4) arithmetic logic unit (ALU), and (5) integer register file (IRF) [7]. Every unit is composed of several modules and the detailed information of each module is listed in Table 1.

The target CMT is exercised with two validation test programs from the OpenSPARC T2 package as described in Table 2 [8]. LDST_ATOMIC.S is memory intensive, while IFU_BASIC_EX_RAW.S is CPU-intensive (abbreviated as LDST and EXU). The CMT is in one core one thread (1c1t) configuration, as the multicore configuration is left for future work.

### 2.2. Fault Injection Framework

**Figure 1:**
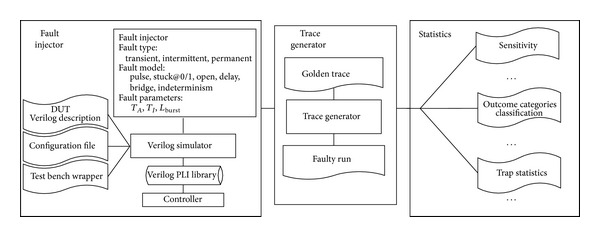
PFIT (Verilog PLI based fault injector) framework.

A fault injection framework, namely, Verilog PLI based fault injector (VPFIT), was designed based on Synopsis VCS to facilitate this work. The overall architecture of VPFIT is depicted in Figure 1, including fault injector, trace generator, and statistics. A series of programming language interface (PLI) tasks, such as Inject_TransFault, Inject_PermFault, and Inject_IntermFault, besides some attendant PLI tasks, including Test_ExecTime, were deliberately designed.

The key features of the VPFIT include (1) automation of injections into the Verilog description of the target CMT, (2) support different fault types (e.g., transient, intermittent, and permanent faults), (3) a variety of fault models (e.g., pulse, stuck-at, open, indeterminism, bridge, and delay in NET versus bit-flip and stuck-at in REG), (4) different fault parameters (e.g., *L*_burst_, *T*_*A*_, and *T*_*I*_ for intermittent faults), (5) automation of trace generation and data collection, and (6) a variety of back-end scripts for analysis and statistics (e.g., classification into outcome categories, computation of sensitivity, and trap statistics).

As the purpose of this work is to characterize the susceptibility indices to intermittent faults for the dependability enhancement strategy at an early design stage, a Verilog description of the target CMT which is independent of implementation and process technology is adopted. The swat-sim like hierarchical simulation is left for future work [7].

### 2.3. Fault Injection Method

On behalf of the fixed location characteristics, intermittent faults are injected into each module of a unit (altogether thirteen modules in this work). To characterize the effects of intermittent faults, transient faults and permanent faults are injected* correspondingly* as well as a reference index.

For each* trial* (fifty fault injections), transient faults are first injected to generate a random template of the fault sites. Then, intermittent faults (and permanent faults) are injected according to the specific configuration for the trial. Fault site includes the following information: module ID, object type (NET or REG), object ID, faulty bit, and the fault injection instant (*T*_inject_) which is randomly chosen from the total execution cycles of the golden trace. For each fault injection instance, only one fault is injected and the workload runs to completion.

According to the object type of the fault site, for example, NET or REG, different fault models are adopted correspondingly for transient faults, intermittent faults, and permanent faults, as listed in Table 3.

For transient faults, a pulse of a duration randomly generated from the [0.01*T*–0.1*T*] interval is applied to NET, while the bit-flip fault model is applied to REG.

For permanent faults, a fault model randomly chosen from the stuck@0/1, indeterminism, and open is applied to NET without drawback until the end of the simulation run, while the stuck@0/1 is applied to REG.

For intermittent faults, a fault model randomly chosen from the pulse, indeterminism, and open is applied to NET, while the bit-flip model is applied to REG.

The fault parameters *T*_*A*_ and *T*_*I*_ are defined according to the uniform distribution function at the ranges [0.01*T*–0.1*T*], [0.1*T*–1*T*], and [1*T*–10*T*], respectively [9].

Here, *T* designates the clock cycle of the target CMT (1 ns in this work) and the smallest simulated time granularity is 1 ps. The parameter *L*_burst_ is specified as two, four, and eight [9].

The combination of fault site, fault model, and fault parameters (e.g., *T*_*A*_, *T*_*I*_, and *L*_burst_ for intermittent faults) constitute a configuration.

For each module under a specific configuration, fifty injection instances constitute a trial and seven trials constitute a* champion*. After a fault injection champion, the back-end statistics are collected. Overall a total of 81,900 simulation runs are performed (350 injections ∗ 13 modules ∗ 3*L*_burst_ ∗ 3*T*_*A*_ ∗ 2 workloads) for intermittent faults and 18,200 runs for permanent and transient faults.

## 3. Sensitivity and Vulnerability Characteristics

Sensitivity is defined as the percentage of faults in an object (NET or REG) pertaining to a given unit or module that results in processor architectural state mismatch [5, 10]. In this section, the target CMT is exercised with two workloads featuring memory intensive and CPU-intensive, respectively, and comprehensive fault injections are conducted to make a thorough investigation of the vulnerability characteristics by using sensitivity metrics.

### 3.1. Sensitivity at Unit Level

Table 4 provides (1) the sensitivity of NET and REG per unit, (2) the sensitivity of transient, permanent, and in particular, intermittent faults for each configuration (the combination of *L*_burst_ and *T*_*A*_, where *L*_burst_ is equal to two, four, and eight, and *T*_*A*_ ranges from the intervals of [0.01*T*, 0.1*T*], [0.1*T*, 1*T*], and [1*T*, 10*T*], resp.).

Taking the EXU workload, for example, analysis of the data leads to several conclusions.

*First*, there is clear evidence that for transient faults the sensitivity of NET (on average 1.1% with a random fault duration ranging from 0.01*T* to 0.1*T* under pulse fault model) is not negligible, even though this figure is five times smaller than the sensitivity of REG (5.5% on average). To the contrary, for permanent faults the sensitivity of NET is 1.59 times higher than that of REG (25.8% versus 16.2%) except for IRF unit.

*Second*, the different configurations of fault parameters *T*_*A*_ and *L*_burst_ ([0.01*T*, 0.1*T*], [0.1*T*, 1*T*], and [1*T*, 10*T*] and two, four, and eight) simulate the exacerbation of wear-out process. On the whole, the sensitivity for a specific configuration of *L*_burst_ increases with respect to the *T*_*A*_, while for a specific *T*_*A*_ sensitivity increases with *L*_burst_. Note that discrepancies exist under some configurations. In-depth analysis reveals that the randomly generated fault models between corresponding fault types (transient, permanent, and intermittent faults) become the leading factor, and the difference between randomly selected fault sites between trials becomes another factor.

*Third*, for units responsible for control, the sensitivity of NET grows more sharply than that of REG as *L*_burst_ and *T*_*A*_ increase, indicating the need for a protection scheme to be employed. To further identify the sphere of protection, an in-depth analysis at a finer granularity—module level—was performed.

### 3.2. Sensitivity Breakup per Unit

A detailed breakdown of the sensitivity per module for EXU and LDST workload is described, respectively, in Tables 5 and 6.

Note, there are three REG objects in PKU_PCK module, but its sensitivity is zero. In-depth analysis reveals that two objects are concerned with scan chain which is disabled, and the other is intrinsic to logical masking of single bit fault. The collected data lead to several important conclusions providing valuable susceptibility indices for the dependability enhancement strategy.

*First*, the impact of fault parameters to sensitivity at the module granularity follows a similar trend as described in the previous section, that the sensitivity for a specific *L*_burst_ increases with respect to *T*_*A*_, while that for a specific *T*_*A*_ increases with *L*_burst_.

*Second*, although the two workloads have different features, a similar trend of the impact of intermittent faults is revealed, and, in particular, the common highly sensitive modules are identified. This corroborates that susceptible characteristics do not vary with workloads, and thus sensitivity can provide valuable information for dependability enhancement strategy [5].

Sensitivity metrics under two workloads reveal that the following modules become the vulnerable bottlenecks for intermittent faults, as listed in Table 7.

Pick unit(**PKU**) is a representative unit in the target CMT which is highly sensitive to intermittent faults, wherein the module of** PKU**_**PKD** in charge of error detection and checking and** PKU**_**SWL** implementing the state machine becomes the bottlenecks. Taking the EXU workload, for example, the sensitivity of PKU_PKD is 24.9% for NET in B2_0.1*T*–1*T* configuration versus 12% for PKU_SWL for REG in B2_0.01–0.1*T* configuration.

In target CMT,** IRF** is well protected from transient faults by ECC module. Whereas, data show that both NET and REG in EXU_IRF module are highly sensitive to intermittent faults with *T*_*A*_ in [1*T*, 10*T*] configuration. In addition, the REG in EXU_RML, a module in charge of register management, is highly sensitive to transient faults, indicating a scheme to protect it from not only intermittent faults but also transient faults. Moreover, the NET of the following modules is highly sensitive: IFU_AGC in AGEN and DEC_DEL in decoder. The REG of the EXU_EDP in ALU is vulnerable as well.

*Thirdly*, above all the collected data press for a protection scheme which can not only cover all of the highly sensitive modules across a variety of units, including PKU, AGEN, decoder, IRF, and ALU, but is also general enough to protect both the NET and REG object types from transient and intermittent faults. When taking into account design and verification complexity, previous approaches which either target a specific unit or aim at some particular parts of the processor are no longer viable [5, 8, 11–14].

Hence, a more general and light-weight method at arch-level, which is not only across different fault types (transient, permanent, and intermittent faults) but also independent of various modules (as listed in Table 7), is a better choice.

## 4. Dependability Enhancement Strategy

### 4.1. Outcome Categories

The fault injection outcome categories are outlined as follows: dead lock (*DLock*), invalid packet request (*IPacket*), short execution (Short), incomplete execution (*IEXE*), bad trap (*BadTrap*), and latent (*Latent*). The detailed description of each category is listed in Table 8.

**Figure 2:**
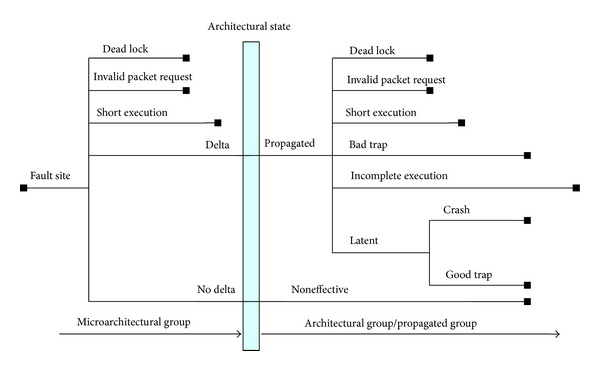
Outcome categories: microarchitectural level groups versus propagated groups.

Figure 2 depicts fault propagation from the fault site through processor architectural state to the application. Through latency analysis, two groups are differentiated:* microarchitectural* group and* propagated* group. Analysis shows that some categories, including* DLock*,* IPacket*, and* Short*, falls into both groups, denoted as *μ* and *p* in Table 8. Note that all the results presented here assume that the probability of the occurrence of intermittent faults for each module is equal.

### 4.2. Dependability Enhancement Strategy

Experimental results of the* u-architectural* and* propagated* groups, respectively, for NET and REG under LDST workload are listed in Table 9. The result of the EXU workload is similar which, due to space constraints, is omitted.

In-depth analysis shows that the following categories lead to* SDC events: DLock, IPacket, IEXE, Short*, and* BadTrap*, as depicted in “*u*-SDC/*p*-SDC events” column.

An alarming statistic is observed for the* u*-architectural group in which the outcome of NET primarily falls into* IPacket*, while that of REG mainly falls into* DLock* event.

Covering as many SDC events as possible is of utmost importance for the dependability enhancement strategy. In-depth analysis reveals that the* DLock* and* IPacket* are detectable symptoms. Data in the “detectable symptoms” column show that for NET the two events contribute to the majority of the* u*-architectural group with a percentage of about 79.0, 71.8, 53.1, 8.9, and 25.4 out of 79.6, 72.4, 67.1, 9.1, and 26.7, respectively, for PKU, AGEN, decoder, ALU, and IRF (80.8, 83.3, 57.6, 25.5, and 29.4 out of 81.0, 83.8, 58.1, 27.4, and 34.6 for EXU workload). This implicates a light-weight protection scheme to contain these two kinds of events as detectable symptoms.

For the propagated group, analysis shows that a simple watchdog can be deployed to cover the* IEXE* event. Thus, the proposed arch-level dependability enhancement strategy can be further improved to contain not only core/strand status and crossbar event but also application running status (*DLock* and* IPacket* versus* IEXE*) as detectable symptoms.

After detailed fault injections, the SDC rate is listed in the SDC′ and SDC^″^ columns in Table 10 after incorporating u-architectural and application level symptoms, respectively. Data demonstrate that by incorporating the* u*-arch level detectable symptoms (*DLock* and* IPacket*) the SDC rate reduces from 6.3% to 0.7% for NET versus 1.3% to 0.2% for REG for LDST workload. By incorporating another application level symptom, namely, IEXE, further SDC decrease is acquired, demonstrating the efficacy of the proposed arch-level dependability enhancement strategy against intermittent faults.

All in all, the above analysis provides a valuable use for reference that the following events, core or strand running status and core-memory interface or crossbar event (*DLock* and* IPacket* in this paper), can be alternatives of arch-level symptoms of hardware faults across a variety of modules for the units under test. Application running status (*IEXE*) can be considered as another symptom to refine strategy.

## 5. Effects of Traditional Protection Schemes to Intermittent Faults

Experimental results demonstrate that 6.5% of traps has triggered out of the manifested symptoms.

In terms of the dependability enhancement strategy, it is impossible to overlook the capability of traditional protection schemes to intermittent faults. In this section, a quantitative study is made on the effect of traditional protection schemes to intermittent faults, demonstrating the necessity of taking into account this factor for the dependability enhancement strategy [6].

In the target CMT, sequential logic is usually protected by traditional schemes such as ECC or parity; besides they are typically concerned with some attendant trap(s) to facilitate protection. The detailed breakdown of traps to each outcome category (*Latent, Incomplete EXEcution, Bad Trap, InvalidPacketRequest,* and* DeadLock*) is depicted in Table 11, showing that majority of traps (99.5%) originate from the propagated group owing to fault propagation.

### 5.1. Protection Effects Quantitative Study

Table 12 describes the effect of traditional protection scheme to intermittent faults for NET and REG, respectively: (1) the priority metric is normalized as the number of occurrence of traps per six champions (2100 injections) expressing a relative weight, (2) the fault coverage and recovery rate for each module, and (3) the contribution of various trap types per module by descending priority. The result of LDST is similar with that of EXU except for some remarkable load/store characteristics, which, due to space constraints, is omitted.

As expected, the parity and ECC is more effective for REG than for NET with an overall priority of 268.9 versus 97.3, and the average fault coverage and recovery rate for REG versus NET (13.9% and 99.2% versus 3.7% and 93.5%) are higher.

For REG, the protection capability for decoder, IRF, and AGEN is expressed by a relative priority of 110.0, 92.4, and 66.5, respectively. However, there is no protective effect for PKU and ALU with the priority of zero.

For net, the priority of 58.6, 16.4, 10.3, 9.0, and 3.1 demonstrates the protection capability for the units PKU, AGEN, decoder, ALU, and IRF, respectively. Of all the modules, PKU_PKD is protected best with a relative priority of 47.3.

The average fault coverage for NET is only 3.7%. However, once an intermittent fault is covered, the traditional scheme is effective with a recovery rate of nearly 100% except ALU and IRF. For ALU, the fault coverage is only 2.3% with the recovery rate of about 22.2%, while IRF is 0.6% versus 66.7%, respectively, indicating the need to protect the logic in ALU and IRF from intermittent faults.

The contribution of different trap types per module is quantitatively described by a relative priority. Data show that, for NET, of all the trap types 0 × 10 takes the majority contribution of about 88% (86.4/97.3). On the contrary, for REG, trap types 0 × 10, 0 × 29, 0 × 0*a*, 0 × 20, and 0 × 11 together contribute nearly 83% (223.9/268.9). This indicates that 0 × 10 fatal trap is of utmost importance to protect both the NET and REG, while other trap types, such as 0 × 29, 0 × 0*a*, and 0 × 20, are vital to protect REG from intermittent faults.

### 5.2. Discussions

The above analysis leads to several prospects for the intermittent faults dependability enhancement strategy.

*First*, for the traditional protection scheme the coverage rate of 3.7% versus 13.9% on average for NET and REG reinforces the advocate of an enhancement strategy to be deployed to counter intermittent faults. The recovery rate of 93.5% versus 99.2% for NET and REG attests the protection effect of traditional scheme to intermittent faults, demonstrating the necessity of taking into account this factor for dependability enhancement.

*Second*, in-depth analysis shows that a simple watchdog can be deployed to cover the* IEXE* event. Thus, the arch-level strategy proposed can be further improved to contain not only core/strand status and crossbar event, but also application running status (*DLock* and* IPacket* versus* IEXE* in this paper) as detectable symptoms. Preliminary estimation shows that on average 0.1% of SDC decrease is acquired for NET across all the units, including AGEN, PKU, decoder, ALU, and IRF under LDST test bench.

*Third* and* last*, we are convinced that the trap would be a promising symptom for fault diagnosis or fault prediction, providing valuable information for architects to further refine the dependability strategy, which is the focus of our future work.

## 6. Related Work

Comprehensive fault injections have been conducted to characterize the effects of transient faults on processors. As semiconductor technology scales into the nanometer regime, a resurgence of interest in intermittent faults has come forth in recent years.

Generally, intermittent faults are assumed to be the prelude of permanent faults. In contrast to transient faults due to single-event upset (SEU), intermittent faults are related to irreversible physical defects in the circuit. These defects can be produced either in the design/manufacturing process or during the normal operation. In the case of normal operation produced defects, a series of wear-out mechanisms can occur in long term perspective, initially revealing as intermittent faults until finally developing into a permanent fault [2]. The SOFs (Source Of Failures) of intermittent faults can be categorized as follows.

Design or manufacturing defects constitute one of the most important SOFs. Residues, process variations, or infant mortality provoked by manufacturing processes, together with design defects, aggravate the situation.

Aging or in-progress wear out becomes another SOF. Complex wear-out mechanisms, such as time dependent dielectric breakdown (TDDB), negative bias temperature instability (NBTI), electromigration (EM), stress migration (SM), and thermal cycling (TC) in packages, are expected to become more frequent in the nanometer regime. Devices typically do not fail suddenly but display intermittent behavior for a period of time beforehand and finally evolve to permanent faults.

Environmental triggers are the inducements for intermittent faults. Continuous shrinking of device feature size due to device scaling leads to increasing susceptibility to various inducements, such as PVT variation, increased cross-talk, and environmental interferences, and so forth.

Above all, the intermittent faults are expected to be an austere challenge of VLSI circuits in the nanometer regime, especially for multi-core in future technologies [15–23].

Accordingly, the computer community commenced to explore the impact of intermittent faults [24, 25]. Rashid et al. made a preliminary study of intermittent faults propagation in application, furthered by Wei et al. [26, 27]. Gracia evaluated the effects of intermittent faults on an embedded system [6, 28]. In contrast to previous work targeting an embedded system or a microcontroller, the UltraSPARC CMT processor is used as a case study in this paper to characterize intermittent faults.

Pan et al. proposed intermittent faults vulnerable factor (IVF), a metric similar to AVF, to estimate the susceptibility of typical sequential units in a processor to intermittent faults [29]. Kim and Somani advocated the sensitivity metric at RTL or lower levels [5]. Saggese et al. made a thorough study of the susceptibility of a superscalar processor to transient faults with the sensitivity metric [10]. Instead of a superscalar, sensitivity metric is adopted to characterize intermittent faults for a CMT; then a protection strategy is proposed in this paper. Experimental results of this paper corroborate Kim's analytic findings that the susceptible characteristics do not vary with workloads on behalf of the sensitivity metrics [5].

Data in this work demonstrate that previous protection schemes targeting a specific unit or some particular parts of a processor are no longer viable [11–14]. Accordingly, an arch-level dependability enhancement strategy, which is not only independent of fault types (intermittent, transient, and permanent faults) but is also applicable across various sensitive modules, is put forward and its potential is evaluated.

## 7. Conclusions

To the best of our knowledge, we are the first to use SPARC T2 processor as a case study to characterize the effects of intermittent faults at register transfer level (RTL) and a dependability enhancement strategy is proposed.

*First*,* sensitivity* evaluation demonstrates that susceptible characteristics do not vary with workloads, and the similar trend of the effect of intermittent faults is revealed and the common sensitive modules are identified.

*Second*, a quantitative study of traditional protection scheme to intermittent faults is made on behalf of the contribution of each trap type, reinforcing the advocate of an enhancement strategy to be deployed to counter intermittent faults while demonstrating the necessity of taking into account this factor for dependability strategy.

*Third*, a thorough breakdown of outcome categories provides a valuable use for reference that the following events, core, or strand status and core-memory interface events (*DLock* and* IPacket* in this paper) can be candidates of arch-level symptoms, whilst workload status (*IEXE*) can be application level symptom to refine the strategy. Data demonstrate that by incorporating arch-level symptoms (*DLock* and* IPacket*) the SDC reduces from 6.3% to 0.7% for NET versus 1.3% to 0.2% for REG. With the additional application level symptom (*IEXE*), further SDC decrease is acquired demonstrating the efficacy of the proposed dependability enhancement strategy for intermittent faults. Thus a general strategy can outline that core/strand running status and crossbar events can be candidates of arch-level symptoms, and workload status can be used as application symptoms to refine the strategy.

## Figures and Tables

**Table 1:** Representative units and corresponding modules under investigation in the target CMT.

Functional Blocks	Unit	Module	Number of NETs	Number of REGs	Description
Control	AGEN	IFU_AGD	3731	122	Address generation
		IFU_AGC	2388	62	AGD control logic
	PKU	PKU_PKD	70	0	Pick error detection or checking
		PKU_PCK	136	3	Pick control logic
		PKU_SWL	2144	78	Thread finite state machines
	Decoder	DEC_DED	415	13	Decode, error detection and checking
		DEC_DCD	184	0	Decoding logic
		DEC_DEL	2411	29	Decode control logic

Execution	ALU	EXU_EDP	2161	30	ALU data path
		EXU_ECT	1381	22	ALU control logic

Storage	IRF	EXU_IRF	329	14	Integer register file
		EXU_ECC	479	11	ECC generation
		EXU_RML	1742	52	Register management logic

**Table 2:** Test benches description.

Test bench	Number of inst.	ALU ops.	Atomic Mem. ops.	Control transfer ops.	Misc. ops.	Descriptions
IFU_BASIC_EX_RAW.S	1194	1178	8	3	8	ALU ops to verify EXU
LDST_ATOMIC.S	363	265	87	3	8	ld./st. ops to verify LSU

**Table 3:** Fault injection method.

Object Type	Transient Faults		Intermittent Faults			Permanent Faults	
	Model	Duration	Model	Duration	Burst Length	Model	Duration
NET	pulse	[0.01*T*–0.1*T*]	pulse	[0.01*T*–0.1*T*]	2/4/8	stuck@0/1	∞
				[0.1*T*–1*T*]			
				[1*T*–10*T*]			
			indeterminism	[0.01*T*–0.1*T*]	2/4/8	indeterminism	∞
				[0.1*T*–1*T*]			
				[1*T*–10*T*]			
			open	[0.01*T*–0.1*T*]	2/4/8	open	∞
				[0.1*T*–1*T*]			
				[1*T*–10*T*]			

REG	Bit-flip	[0.01*T*–0.1*T*]	bit-flip	[0.01*T*–0.1*T*]	2/4/8	stuck@0/1	∞
				[0.1*T*–1*T*]			
				[1*T*–10*T*]			

**Table 4:** Sensitivity per unit (%).

Units	Object type	Transient faults	Intermittent faults									Permanent faults
			B2_	B2_	B2_	B4_	B4_	B4_	B8_	B8_	B8_	
			0.01–0.1	0.1–1	1–10	0.01–0.1	0.1–1	1–10	0.01–0.1	0.1–1	1–10	
PKU	NET	1.2	1.6	12.1	19.4	3.7	15.1	23.7	4.2	18.1	22.9	23.5
	REG	10.3	11.2	13.1	12.2	12.2	14.0	17.8	12.2	15.9	17.8	17.8
AGEN	NET	1.2	1.7	6.5	15.3	2.3	10.9	18.6	5.4	12.3	23.6	23.9
	REG	1.4	1.1	1.4	7.0	1.7	3.7	10.1	2.5	6.6	11.0	10.5
Decoder	NET	1.8	1.2	9.8	19.0	5.4	13.6	22.4	6.2	15.3	24.7	22.5
	REG	4.2	1.6	2.4	8.0	4.6	2.3	11.7	3.7	5.1	12.0	10.5
ALU	NET	0.9	1.3	4.2	12.2	2.0	6.8	20.1	2.8	9.2	25.8	41.4
	REG	4.6	4.2	4.3	13.5	2.6	7.0	19.2	5.5	8.7	24.3	23.6
IRF	NET	0.6	0.4	6.7	12.5	3.1	8.8	15.3	3.1	10.7	17.6	14.3
	REG	8.2	7.9	8.6	13.9	8.3	10.0	21.6	7.9	13.1	22.6	15.3

**Table:** (a) For NET (%)

Units	Module	Transient faults	Intermittent faults									Permanent faults
			B2_	B2_	B2_	B4_	B4_	B4_	B8_	B8_	B8_	
			0.01–0.1	0.1–1	1–10	0.01–0.1	0.1–1	1–10	0.01–0.1	0.1–1	1–10	
PKU	PKU_PCK	0.0	0.8	4.4	8.0	2.4	5.6	8.8	3.2	7.6	8.8	8.0
	PKU_PKD	3.1	3.4	24.9	41.1	6.9	25.4	47.7	12.3	33.4	56.3	53.7
	PKU_SWL	1.2	1.6	12.1	19.4	3.6	15.4	23.9	4.0	18.2	22.7	23.5

AGEN	IFU_AGD	1.7	1.3	4.6	14.3	0.8	8.9	17.3	4.2	11.4	23.2	23.2
	IFU_AGC	0.5	2.3	9.6	16.9	4.6	14.2	20.5	7.3	13.7	24.2	25.2

Decoder	DEC_DED	0.4	1.2	4.9	16.2	1.6	4.5	20.2	2.0	9.7	25.9	27.1
	DEC_DCD	0.0	0.0	2.0	8.0	5.7	7.7	8.0	0.3	1.1	4.6	6.6
	DEC_DEL	2.2	1.3	11.3	20.3	6.1	15.6	23.8	7.4	17.3	26.0	22.9

ALU	EXU_ECT	1.7	1.3	5.9	13.5	3.0	8.4	17.3	3.8	9.3	24.6	27.8
	EXU_EDP	0.4	1.3	3.1	11.4	1.3	5.7	21.9	2.2	9.2	26.3	50.0

IRF	EXU_IRF	1.7	2.1	5.2	20.2	3.0	7.7	27.9	4.3	13.7	33.5	33.7
	EXU_RML	0.5	0.0	8.3	12.8	3.7	10.6	14.7	3.7	11.9	16.1	52.6
	EXU_ECC	0.4	0.4	2.1	5.9	0.8	3.4	8.8	0.4	4.2	12.2	13.8

**Table:** (b) For REG (%)

Units	Module	Transient faults	Intermittent faults									Permanent faults
			B2_	B2_	B2_	B4_	B4_	B4_	B8_	B8_	B8_	
			0.01–0.1	0.1–1	1–10	0.01–0.1	0.1–1	1–10	0.01–0.1	0.1–1	1–10	
PKU	PKU_PCK	0	0	0	0	0	0	0	0	0	0	0
	PKU_PKD	—	—	—	—	—	—	—	—	—	—	—
	PKU_SWL	11	12	14	13	13	15	18	13	17	18	18

AGEN	IFU_AGD	0.9	0.9	1.8	7.1	1.8	4.4	10.6	2.7	8.0	11.5	11.5
	IFU_AGC	2.3	1.5	0.8	6.9	1.5	2.3	9.2	2.3	3.8	9.9	8.4

Decoder	DEC_DED	3.9	1.0	1.9	15.5	1.9	5.8	16.5	1.0	10.7	18.4	18.4
	DEC_DCD	—	—	—	—	—	—	—	—	—	—	—
	DEC_DEL	4.2	1.7	2.5	6.7	5.0	1.7	10.9	4.2	4.2	10.9	9.2

ALU	EXU_ECT	3.5	3.5	4.4	12.4	3.5	4.4	14.2	5.3	6.2	18.6	20.4
	EXU_EDP	5.7	4.9	4.1	14.8	1.6	9.8	24.6	5.7	11.5	30.3	27.0

IRF	EXU_IRF	6.0	4.3	9.4	23.1	3.4	9.4	36.8	6.0	16.2	37.6	39.3
	EXU_RML	9.8	9.8	9.8	13.6	10.6	12.1	20.5	9.8	14.4	21.2	11.4
	EXU_ECC	3.6	3.6	1.8	3.6	3.6	0.9	8.0	0.9	2.7	9.8	3.6

**Table:** (a) For NET (%)

Units	Module	Transient faults	Intermittent faults									Permanent faults
			B2_	B2_	B2_	B4_	B4_	B4_	B8_	B8_	B8_	
			0.01–0.1	0.1–1	1–10	0.01–0.1	0.1–1	1–10	0.01–0.1	0.1–1	1–10	
PKU	PKU_PCK	2.6	0.9	3.9	9.2	1.7	7.4	8.7	3.1	5.2	9.6	9.6
	PKU_PKD	1.1	2.9	21.7	38.3	6.6	24.6	48.3	13.1	31.1	56.6	54.6
	PKU_SWL	2.0	4.4	14.1	26.2	5.2	16.9	26.2	9.7	20.2	29.0	33.1

AGEN	IFU_AGD	0.5	1.4	5.6	11.7	2.3	8.5	13.1	6.1	8.5	17.4	26.3
	IFU_AGC	2.1	1.3	11.7	17.9	4.6	14.2	20.0	6.3	15.4	24.6	26.7

Decoder	DEC_DED	0.8	1.6	2.8	9.3	2.4	3.2	13.4	2.0	4.5	19.0	20.6
	DEC_DCD	0.3	0.3	2.0	6.3	0.3	1.4	10.9	0.0	2.9	17.7	27.1
	DEC_DEL	0.9	1.3	12.9	19.3	3.0	14.2	24.0	5.6	14.2	30.0	30.5

ALU	EXU_ECT	0.5	1.8	1.8	9.2	1.4	4.6	11.0	2.8	6.9	15.6	20.2
	EXU_EDP	0.9	0.9	4.1	10.6	1.8	3.2	11.9	1.8	5.5	23.9	39.4

IRF	EXU_IRF	0.4	0.8	5.9	19.5	2.1	8.9	25.4	3.8	11.9	39.0	47.5
	EXU_RML	0.0	1.2	2.8	7.3	1.2	4.5	8.5	2.0	4.5	13.4	10.9
	EXU_ECC	0.0	1.3	1.3	4.3	2.2	1.7	6.1	1.7	3.5	8.2	13.9

**Table:** (b) For REG (%)

Units	Module	Transient faults	Intermittent faults									Permanent faults
			B2_	B2_	B2_	B4_	B4_	B4_	B8_	B8_	B8_	
			0.01–0.1	0.1–1	1–10	0.01–0.1	0.1–1	1–10	0.01–0.1	0.1–1	1–10	
PKU	PKU_PCK	0.0	0.0	0.0	0.0	0.0	0.0	0.0	0.0	0.0	0.0	0.0
	PKU_PKD	—	—	—	—	—	—	—	—	—	—	—
	PKU_SWL	9.8	9.8	9.8	17.6	7.8	12.7	16.7	10.8	14.7	22.5	26.5

AGEN	IFU_AGD	0.0	0.0	1.5	3.6	0.7	0.7	4.4	0.0	5.1	8.0	7.3
	IFU_AGC	0.9	0.9	0.0	3.6	0.9	2.7	4.5	1.8	2.7	7.3	12.7

Decoder	DEC_DED	2.9	1.9	1.9	19.4	1.0	3.9	22.3	3.9	13.6	35.0	24.3
	DEC_DCD	—	—	—	—	—	—	—	—	—	—	—
	DEC_DEL	1.7	2.6	6.0	6.0	3.4	0.9	12.8	1.7	7.7	12.8	16.2

ALU	EXU_ECT	0.8	0.0	1.5	3.0	0.0	3.8	3.8	0.8	3.0	3.8	12.9
	EXU_EDP	0.8	2.3	3.8	5.3	3.8	2.3	11.4	0.0	6.1	20.5	28.8

IRF	EXU_IRF	4.4	2.6	8.8	19.3	3.5	11.4	29.8	5.3	21.9	39.5	51.8
	EXU_RML	4.9	4.9	4.9	5.8	4.9	4.9	9.7	4.9	5.8	11.7	4.9
	EXU_ECC	0.0	1.7	0.8	0.8	0.8	1.7	1.7	0.0	2.5	2.5	1.7

**Table 7:** Module level vulnerable bottlenecks.

Object type	Units	Modules	Workloads	
			EXU	LDST
NET	PKU	PKU_PKD	√	√
		PKU_SWL	√	√
	IRF	EXU_IRF	√	√
	AGEN	IFU_AGC	√	√
	Decoder	DEC_DEL	√	√

REG	PKU	PKU_SWL	√	√
	IRF	EXU_IRF	√	√
		EXU_RML	√	√
	ALU	EXU_EDP	√	√

**Table 8:** Outcome categories description.

Outcome categories	Groups	Description
Dead lock	*u* and p	All threads no activity for 3000 cycles, global timeout
Invalid packet request	*u* and p	An invalid request packet is provoked and the processor is in idle state because of an invalid request packet
Short	*u* and p	Thread completes prematurely before the expected execution time
Incomplete execution	p	Thread is not complete in the expected time (normal execution time + 10% extra time margin)
Bad trap	p	Incorrect thread execution result
Latent	p	Execution result of the thread is correct without a crash

*u*: *u*-architectural group, p: propagated group.

**Table:** (a) Detectable symptoms breakdown for NET (%)

Units	u-architectural group						Propagated group								
	u-SDC events					(%)	Non-SDC events	p-SDC events							(%)
	SDC	Detectable symptoms						SDC		Detectable symptoms				
	Short	Dead lock	IPacket				Latent	Bad trap	Short	Dead lock	IPacket	IEXE		
PKU	0.5	0.9	78.1	79.0	79.6	79.6	17.3			0.8	2.3		3.1	3.1	20.4
AGEN	0.6	2.4	69.4	71.8	72.4	72.4	18.7			0.6	8.3		8.9	8.9	27.6
Decoder	14.1	0.0	53.1	53.1	67.1	67.1	32.1			0.3	0.5		0.8	0.8	32.9
ALU	0.3	0.5	8.4	8.9	9.1	9.1	84.1			1.0	4.1	1.8	6.8	6.8	90.9
IRF	1.3	0.9	24.5	25.4	26.7	26.7	69.0		0.2	1.8	2.4		4.2	4.4	73.3

**Table:** (b) Detectable symptoms breakdown for REG (%)

Units	u-architectural group						Propagated group								
	u-SDC events					(%)	Non-SDC events	p-SDC events							(%)
	SDC	Detectable symptoms						SDC		Detectable symptoms				
	Short	Dead lock	IPacket				Latent	Bad trap	Short	Dead lock	IPacket	IEXE		
PKU	0.7	71.1	0.7	71.9	72.6	72.6	27.4								27.4
AGEN		14.3		14.3	14.3	14.3	84.8			1.0			1.0	1.0	85.7
Decoder							98.5				0.8	0.8	1.5	1.5	100
ALU							100.0								100
IRF							100.0								100

**Table:** (a) SDC for NET (%)

Functional Blocks	Unit	LDST testbench			EXU testbench		
		SDC	SDC′	SDC′′	SDC	SDC′	SDC′′
Control	PKU	15.6	0.7	0.6	14.1	0.1	0.1
	AGEN	9.2	0.2	0.1	8.8	0.1	0.1
	Decoder	5.8	0.8	0.7	5.9	1.2	1.2
Execution	ALU	4.1	1.1	1.0	1.5	0.2	0.0
Storage	IRF	3.2	0.8	0.7	2.6	0.1	0.1
	Average	7.6	0.7	0.6	6.6	0.3	0.3

**Table:** (b) SDC for REG (%)

Functional Blocks	Unit	LDST testbench			EXU testbench		
		SDC	SDC′	SDC′′	SDC	SDC′	SDC′′
Control	PKU	4.1	0.9	0.9	5.3	0.1	0.1
	AGEN	0.2	0.1	0.0	0.7	0.0	0.0
	Decoder	2.1	0.1	0.0	0.1	0.1	0.0
Execution	ALU	0.1	0.0	0.0	0.0	0.0	0.0
Storage	IRF	0.1	0.1	0.0	0.0	0.0	0.0
	Average	1.3	0.2	0.2	1.2	0.0	0.0

**Table 11:** Trap distributions.

Outcome categories	% of traps
Latent	63.4%
Incomplete execution	33.9%
Bad trap	2.2%
Propagated	99.5%

Invalid packet request	0.2%
Dead lock	0.2%
Sum	100%

**Table:** (a) For NET

Units	Module	Priority	Trap contribution	Capability	
			Trap type and relative priority	Coverage	Recovery rate
PKU	PCK	10.3	0x10 10.3	8.9%	100.0%
	PKD	47.3	0x10 47.3	8.1%	100.0%
	SWL	0.9	0x10 0.9	0.3%	100.0%
	Average	58.6	0x10 58.6	6.4%	100.0%

AGEN	AGD	7.9	0x10 7.9	3.9%	100.0%
	AGC	8.5	0x10 4.3 0x0a 2.1 0x71 2.1	3.2%	100.0%
	Average	16.4	0x10 12.1 0x0a 2.1 0x71 2.1	3.5%	100.0%

Decoder	DED	1.9	0x0a 0.9 0x3e 0.9	0.9%	100.0%
	DCD	5.3	0x10 4.7 0x04 0.7	6.1%	100.0%
	DEL	3.0	0x10 2.0 0x04 1.0	1.0%	100.0%
	Average	10.3	0x10 6.7 0x04 1.7 0x0a 0.9 0x3e 0.9	2.0%	100%

ALU	ECT	4.9	0x10 3.9 0x1fa 1.0	2.4%	20.0%
	EDP	4.1	0x10 4.1	2.1%	25.0%
	Average	9.0	0x10 8.0 0x1fa 1.0	2.3%	22.2%

IRF	IRF	1.0	0x10 1.0	0.4%	0.0%
	RML	1.1	0xc0 1.1	0.6%	100.0%
	ECC	1.0	0x29 1.0	1.1%	100.0%
	Average	3.1	0xc0 1.1 0x10 1.0 0x29 1.0	0.6%	66.7%

NET		97.3	0x10 86.4 0xa 3.1 0x71 2.1 0x04 1.7 0xc0 1.1 0x29 1.0 0x1fa 1.0 0x3e 0.9	3.7%	93.5%

**Table:** (b) For REG

Units	Module	Priority	Trap contribution	Capability	
			Trap type and relative priority	Coverage	Recovery rate
PKU	PCK	0.0		0.0%	0.0%
	PKD	—	—	—	—
	SWL	0.0		0.0%	0.0%
	Average	0.0		0.0%	0.0%

AGEN	AGD	43.4	0x10 43.4	38.2%	100.0%
	AGC	23.2	0x10 17.8 0x0d 5.3	26.0%	100.0%
	Average	66.5	0x10 61.2 0x0d 5.3	32.4%	100.0%

Decoder	DED	45.3	0x0a 24.9 0x3e 9.1 0x64 4.5	26.7%	95.0%
	DCD	—	—	—	—
	DEL	64.7	0x10 23.5 0x20 21.6 0x11 19.6	57.9%	100.0%
	Average	110.0	0x0a 24.9 0x10 23.5 0x20 21.6 0x11 19.6 0x3e 9.1	40.2%	98.1%

ALU	ECT	0.0		0.0%	0.0%
	EDP	0.0		0.0%	0.0%
	Average	0.0		0.0%	0.0%

IRF	IRF	0.0		0.0%	0.0%
	RML	19.4	0x24 19.4	6.8%	100.0%
	ECC	72.9	0x29 72.9	89.7%	100.0%
	Average	92.4	0x29 72.9 0x24 19.4	12.4%	100.0%

REG		268.9	0x10 84.7 0x29 72.9 0x0a 24.9 0x20 21.6 0x11 19.6 0x24 19.4 0x3e 9.1 0xd 5.3	13.9%	99.2%
